# The Degree of Temporal Synchronization of the Pulse Oscillations from a Gain-Switched Multimode Semiconductor Laser

**DOI:** 10.3390/ma10080950

**Published:** 2017-08-15

**Authors:** Kenji Wada, Naoaki Kitagawa, Tetsuya Matsuyama

**Affiliations:** Department of Physics and Electronics, Osaka Prefecture University, Sakai, Osaka 599-8531, Japan; mnch.mai@gmail.com (N.K.); matsu@pe.osakafu-u.ac.jp (T.M.)

**Keywords:** semiconductor laser, gain-switching, multimode oscillation, simultaneous pulse oscillation, terahertz dime-domain spectroscopy, Langevin noise, timing and amplitude jitter

## Abstract

Langevin noise leads to inhibition of the temporal synchronization of the pulse oscillations from a gain-switched multimode semiconductor laser, resulting in the power reduction in optical beat detection. In this paper, the degree of the temporal synchronization of the pulse oscillations was examined by numerically estimating the output energy in THz time-domain spectroscopy (THz-TDS) using multimode semiconductor laser rate equations that include Langevin noise. The degree was estimated to be 95.5% from the ratio of the averaged THz-TDS output energy for the case where Langevin noise was included to that for when Langevin noise was excluded. Therefore, a gain-switched multimode semiconductor laser can be regarded as equivalent to optical pulses oscillating simultaneously in all modes in actual applications including optical beat detection.

## 1. Introduction

Gain-switched semiconductor lasers have been used as compact picosecond optical sources in many applications, such as distance measurements [1,2,3], nonlinear microscope imaging [4,5], and optical communications [6,7]. In many cases, distributed feedback (DFB) lasers are used to meet the requirements of high pulse peak power and/or single-mode (narrow bandwidth) operation. However, the timing jitter in gain-switched multimode semiconductor lasers is much smaller than that in gain-switched single-mode semiconductor lasers, typically by a factor of 3–5 [8,9,10]. Therefore, Fabry–Perot (FP) lasers are also useful because low-jitter, wavelength-tunable, single-mode, gain-switched pulses can be obtained by the injection seeding technique [11,12,13,14]. 

Recently, we have reported that the suppression of the timing jitter in gain-switched FP lasers is caused by a decrease in the coherence time of the amplified spontaneous emission (ASE) based on the broad bandwidth of multimode oscillation. We have also shown that fluctuations in the ASE cause amplitude jitter, instead of timing jitter, in the pulse components of the respective modes [15]. The amplitude jitter disturbs fully simultaneous pulse oscillations among the multiple modes. Estimating the degree of temporal synchronization of the pulse oscillations from a gain-switched multimode semiconductor laser is an interesting subject of study. However, because the amplitude jitter is generally not important in the average detection, estimating the degree has not been given much importance in actual applications except for the field of optical communications.

The situation is different for the case of detecting optical beat signals. A typical example is THz time-domain spectroscopy (THz-TDS) with a multimode laser as an input light source [16,17,18,19]. In this case, the THz-TDS output strongly depends on the degree of temporal synchronization of the light components among the multiple modes. In this paper, we numerically estimate the degree of temporal synchronization of the pulse oscillations from a gain-switched multimode semiconductor laser by simulating the THz-TDS output.

## 2. Multimode Semiconductor Laser Rate Equations

We start by simulating a gain-switched multimode semiconductor laser using the multimode semiconductor laser rate equations, including Langevin noise [15,20,21]:

(1) $${\dot{E}}_{n}\left(t\right)=\frac{1+i\alpha }{2}\left[g_{n}\left(N\right)-\frac{1}{\tau_{p}}+\frac{\beta C_{2}N{\left(t\right)}^{2}}{S_{n}\left(t\right)}+\sqrt{\frac{2\beta C_{2}N{\left(t\right)}^{2}}{S_{n}\left(t\right)\Delta \tau_{c}}}\xi_{Sn}\left(\Delta \tau_{c}\right)+\frac{i2n\delta \omega }{1+i\alpha }\right]E_{n}\left(t\right),$$

(2) $$\dot{N}\left(t\right)=\frac{I\left(t\right)}{eV}-\frac{N\left(t\right)}{T_{1}\left(N\right)}-\sum_{n}\left[g_{n}\left(N\right)S_{n}\left(t\right)+\sqrt{\frac{2S_{n}\left(t\right)\beta C_{2}N{\left(t\right)}^{2}}{\Delta \tau_{c}}}\xi_{Sn}\left(\Delta \tau_{c}\right)-\sqrt{\frac{2N\left(t\right)}{T_{1}\left(N\right)\Delta \tau_{c}}}\xi_{Nn}\left(\Delta \tau_{c}\right)\right],$$

where *E_n_*(*t*), *S_n_*(*t*), and *N*(*t*) are the complex optical electric field and photon density for the *n*th mode, and the carrier density, respectively. *S_n_*(*t*) is equal to *n_r_*^2^*ε*_0_*|E_n_*(*t*)*|*^2^/2*ћω_n_*. The modal gain *g_n_*(*N*) and the carrier density dependent carrier lifetime, *T*_1_(*N*), are given by

(3) $$g_{n}\left(N\right)=\frac{G_{0n}\left(N\left(t\right)-N_{0n}\right)}{\left[1+\varepsilon_{Nn}\left(N\left(t\right)-N_{0n}\right)\right]\left(1+\varepsilon_{S}\sum_{j}S_{j}\left(t\right)\right)},$$

(4) $$\frac{1}{T_{1}\left(N\right)}=C_{1}+C_{2}N\left(t\right)+C_{3}N{\left(t\right)}^{2}.$$

The injected current, *I*(*t*), for gain switching is represented as the sum of a dc-bias current and a sinusoidally modulated current, in the form:

(5) $$I\left(t\right)=I_{dc}-I_{mw}\cos2\pi f_{\mathrm{mod}}t\;\left[I\left(t\right)=0\;for\;I\left(t\right)<0\right].$$

*ξ_Sn_*(≥Δ*τ_c_*) and *ξ_Nn_*(Δ*τ_c_*) are the zero-mean and unit-variance Gaussian distributions, respectively, whose amplitudes are varied every Δ*τ_c_* as step functions [15]. $\xi_{\mathrm{Sn}}$, the differential gain coefficient, *G*_0*n*_, the carrier density at transparency, *N*_0*n*_, and the intrinsic gain saturation coefficient, *ε_Nn_*, for the *n*th-mode are represented by polynomial equations of mode number *n* in Equation (6). The condition *n* = 0 stands for the central mode, which is set to 375 THz (800 nm) by taking into account wavelength matching with a GaAs photoconductive antenna. A negative mode number indicates that the oscillation frequency for the mode of interest is lower than the central frequency, while a positive mode number indicates that it is higher. The coefficients *a_j_*, *b_j_*, and *c_j_* in Equation (6) take values suitable for expressing the gain of an 800-nm semiconductor laser, as listed in Table 1, and are obtained by numerical fitting in the same way as in previous studies [22,23]:

(6) $$G_{0n}=\sum_{j=0}^{14}a_{j}n^{j},\;N_{0n}=\sum_{j=0}^{14}b_{j}n^{j},\;\varepsilon_{Nn}=\sum_{j=0}^{14}c_{j}n^{j},\;\left(-30\leq n\leq 30\right).$$

**Figure 1:**
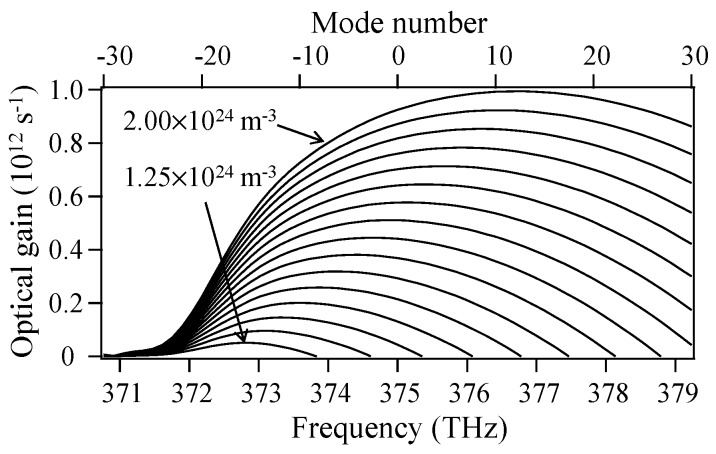
Gain spectra of an 800-nm semiconductor laser calculated using Equations (3) and (6) when the carrier density is varied from 1.25 × 10^24^ m^−3^ to 2.00 × 10^24^ m^−3^ in 0.05 × 10^24^ m^−3^ steps.

Figure 1 shows the 800-nm semiconductor laser gain spectra obtained by varying the mode number *n* in Equations (3) and (6) when *S_j_*(*t*) = 0. The carrier density is set as a running parameter and is varied from 1.25 × 10^24^ m^−^^3^ to 2.00 × 10^24^ m^−^^3^ in 0.05 × 10^24^ m^−^^3^ steps. The notation and values for the other parameters are listed in Table 2.

## 3. Gain-Switched Pulses from a Multimode Semiconductor Laser

The temporal evolution of the variables *E_n_*(*t*) and *N*(*t*) was then numerically evaluated by the fourth-order Runge–Kutta method under the gain switching condition of Equation (5). Repeating the numerical integration produced a regular gain-switched pulse train within five cycles of the modulation period (1*/f_mod_* = 1 ns). The absolute values squared of the Fourier transform of the temporal data of the optical fields provided the power spectrum of the gain-switched pulse. 

**Figure 2:**
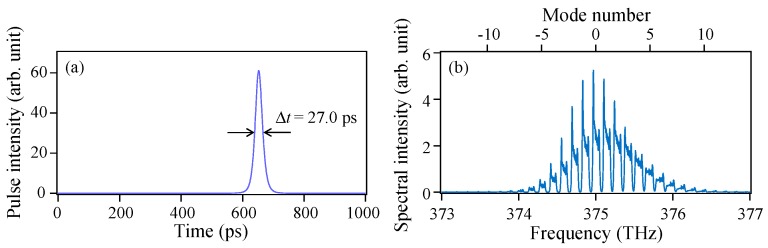
Simulated (**a**) temporal waveform averaged over 1000 single-shot pulses and (**b**) the corresponding power spectrum of the gain-switched pulse when the Langevin noise terms in Equations (1) and (2) are omitted (*ξ_Sn_* = *ξ_Nn_* = 0).

Figure 2 shows (a) a simulated averaged temporal waveform and (b) the corresponding power spectrum of the gain-switched pulse when the Langevin noise terms in Equations (1) and (2) are omitted (*ξ_Sn_* = *ξ_Nn_* = 0). In this ideal case, pulse oscillations occur simultaneously at about 20 modes. Although the spectral shape shown in Figure 2b is unchanged for arbitrary phase relations among multiple modes, the single-shot temporal waveform changes significantly. Therefore, to obtain the averaged temporal waveform shown in Figure 2a, the optical field components of the respective modes must be summed 1000 times under different phase conditions and then the waveform must be low-pass filtered. This coherent addition operation was found to be the same as the incoherent summation of the intensity profiles of the pulse components at the respective modes. Because the former method requires considerable care, we used the latter method to save time. 

**Figure 3:**
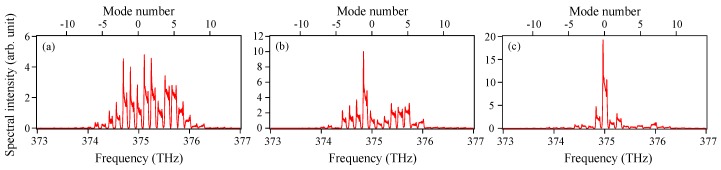
Examples of power spectra of the single-shot gain-switched pulses with Langevin noise included. Langevin noise amplitudes are different for the respective cases of (**a**–**c**).

**Figure 4:**
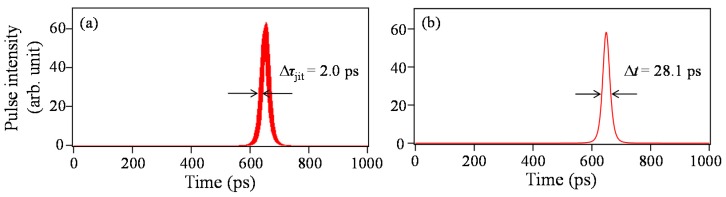
(**a**) Superimposed temporal waveform of 2000 pulses and (**b**) the corresponding averaged waveform with Langevin noise included.

In contrast, in the presence of Langevin noise, the spectral shape changes significantly for every shot, as shown in Figure 3, meaning that the pulse components of about 20 modes oscillate asynchronously. The averaged temporal waveforms for the single-shot spectra obtained by the same method as for Figure 2a are somewhat different from one another due to a residual timing jitter. Figure 4a shows a superimposed pulse waveform consisting of 2000 traces of the averaged temporal waveforms. The residual timing jitter Δ*τ_jit_* in the Figure 4a is estimated to be 2.0 ps, defined as one standard deviation of the HWHM (half width at half maximum) of the optical pulses. Although a multimode oscillation effectively suppresses the generation of timing jitter, a relatively large timing jitter compared with the pulse width remains in Figure 4a because of the small number of oscillating modes. Then, 2000 averaged temporal waveforms are averaged again, as shown in Figure 4b. The waveforms in Figure 4a,b correspond to the raw and averaged pulse traces observed with a sampling oscilloscope, respectively. It is found from a comparison of Figure 2a and Figure 4b that the pulse width broadens to 28.1 ps from 27.0 ps due to the residual timing jitter caused by Langevin noise.

**Figure 5:**
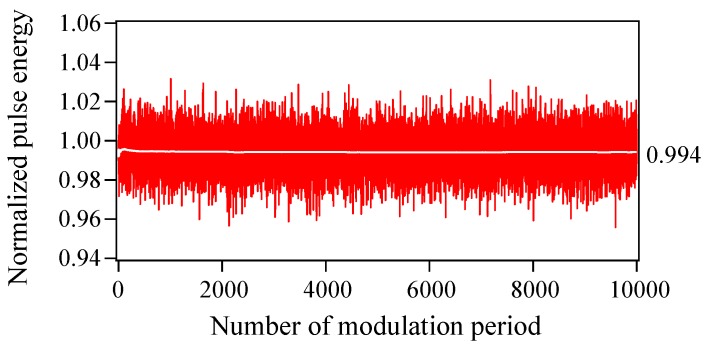
Temporal variation in the pulse energy from 0 to 10,000 times the modulation period with Langevin noise included. The pulse energy is normalized to that in the absence of Langevin noise. The averaged pulse energy is indicated by a white line.

**Figure 6:**
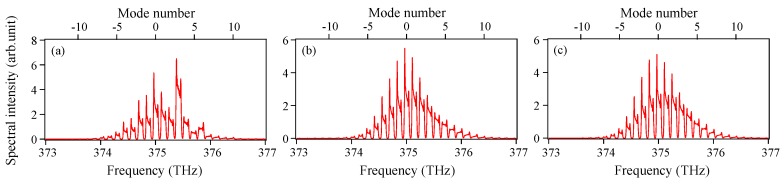
Examples of averaged spectra when (**a**) 5; (**b**) 100; (**c**) 2000 different single-shot spectra are summed, with Langevin noise included.

**Figure 7:**
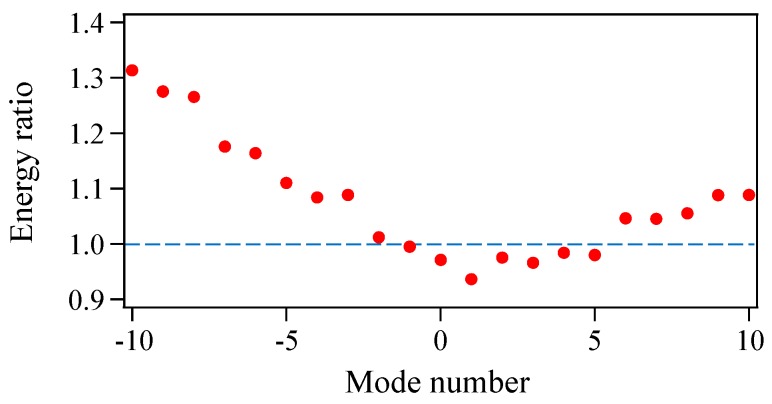
Ratio of pulse energy at each mode in Figure 6c to that in Figure 2b.

Figure 5 shows the temporal variation in the pulse energy from 0 to 10,000 times the modulation period (0–10 μs) in the presence of Langevin noise. The pulse energy ($\propto \underset{n,\omega }{\sum }{\left|{\tilde{E}}_{n}\left(\omega \right)\right|}^{2}$) is normalized to that in the absence of Langevin noise. Although the single-shot pulse energy varies randomly from 0.96 to 1.03, the fluctuations due to Langevin noise are small and the averaged pulse energy converges on an approximately constant value of 0.994 within 2000 modulation periods (2 μs). Figure 6 shows the averaged spectra when (a) 5, (b) 100, and (c) 2000 different single-shot spectra are summed. Corresponding to the result in Figure 5, the spectral shape of the averaged power spectrum in Figure 6c almost matches that in Figure 2b. To examine the effect of Langevin noise in detail, the ratio of the pulse energy at each mode in Figure 6c to that in Figure 2b is plotted in Figure 7. It is found that the ratio is less than 1.0 around the central mode (mode number from −1 to 5) and becomes larger for large values of the absolute mode number. This is because a simultaneous oscillation among multiple modes in the absence of Langevin noise accompanies a relative gain-narrowing compared with the case in the presence of Langevin noise.

## 4. Verification of Simultaneous Oscillation among Multiple Modes Using THz-TDS

**Figure 8:**
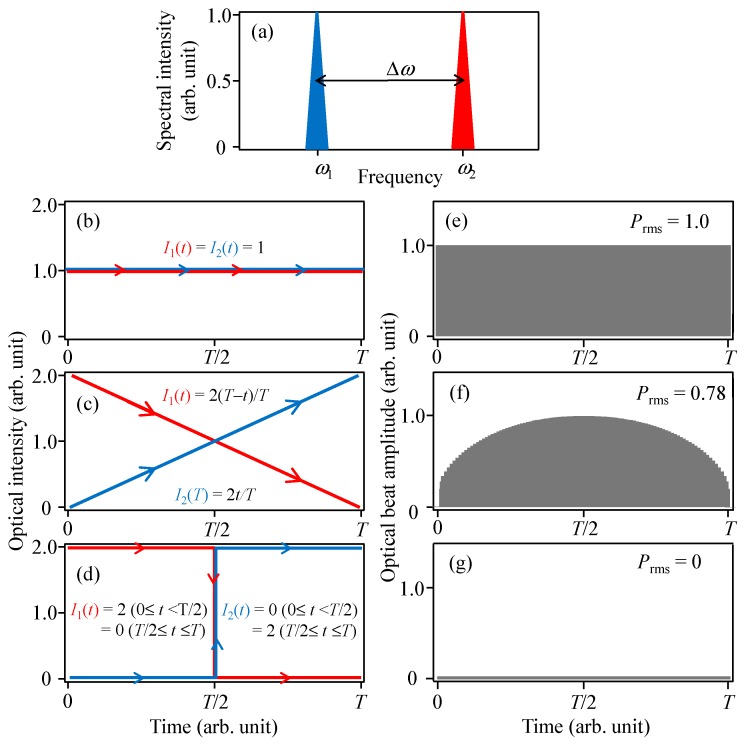
(**a**) Two-mode light spectrum; (**b**–**d**) examples of different input temporal waveforms and (**e**–**g**) corresponding optical beat outputs.

As mentioned, a clear difference between the ideal (Langevin noise excluded) and actual (Langevin noise included) gain-switched multimode semiconductor lasers is whether or not the pulse oscillations at all oscillating modes are simultaneously generated. From the results of Figure 2b and Figure 6c, it is found that there is almost no difference between them when an average detection is carried out and the measurement time is longer than 2000 times the modulation period. However, as described in the Introduction, the situation may be different for the detection of optical beat signals even if the averaged detection process is included. Figure 8 shows examples of optical beat outputs for different input temporal waveforms. Two-mode light having a difference angular frequency of Δ*ω* is assumed as input light in Figure 8a. Figure 8e–g shows the temporal variation of the amplitudes of the optical beat signals for three cases: when the two light intensities are both constant with time (fully simultaneous) (Figure 8b), when one is increased and the other is decreased linearly (partly simultaneous) (Figure 8c), and when one is increased and the other is decreased in a staircase pattern (non-simultaneous) (Figure 8d). The figures show that although the power spectra of the temporal waveforms are nearly the same, the power of the corresponding optical beat signal significantly differs depending on the degree of the synchronization of the temporal waveforms. The root-mean-square (rms) power, *P*_rms_, of the optical beat signal in Figure 8 is given in the following form:

(7) $$P_{rms}=\;K\sqrt{\frac{1}{T}\int_{0}^{T}{\left(\sqrt{I_{1}I_{2}}\cos\Delta \omega t\right)}^{2}dt},$$

where *I*_1_ and *I*_2_ are the intensities of the light components with oscillation angular frequencies *ω*_1_ and *ω*_2_, respectively, and *K* is a constant. When *I*_1_ and *I*_2_ are set to the values shown in Figure 8b–d, and *K* and *T* are set to $\sqrt{2}$ and 2*mπ*/Δ*ω* (*m*: integer), *P*_rms_ is calculated to be 1.0 (Figure 8e), 0.78 (Figure 8f), and 0 (Figure 8g), respectively. The maximum value of *P*_rms_ (*P*_rms_max_) is given in the fully simultaneous condition, and *P*_rms_/*P*_rms*_*max_ can be used as an index, ranging from 0 to 1, to estimate the degree of the temporal synchronization of the two-mode oscillation, and may also be used for multimode oscillation. The optical beat signal using a gain-switched multimode semiconductor laser is obtained in the THz region. Because a direct observation to measure *P*_rms_ in the THz-region is difficult, a THz time-domain spectroscopy (THz-TDS) system is employed to detect THz-waves in actual experiments. We therefore examine the degree of temporal synchronization of the pulse oscillations by numerically estimating the THz-TDS outputs for gain-switched pulse inputs, instead of *P*_rms_. 

**Figure 9:**
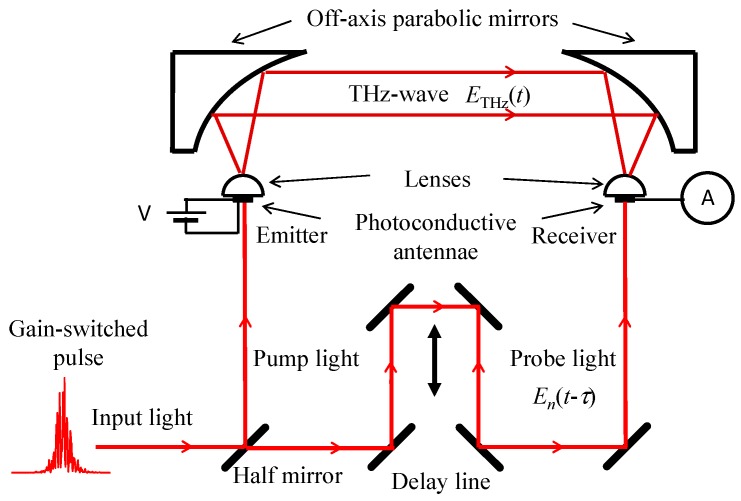
Schematic diagram of THz-TDS (time-domain spectroscopy) system.

A typical THz-TDS system employed as a numerical model is shown in Figure 9. An input gain-switched pulse from a multimode semiconductor laser is divided equally into pump light and probe light through a half mirror. The pump light is focused on a gap between metallic electrodes of one photoconductive antenna to create photo-carriers, *n*(*t*), which is described in the form:

(8) $$n\left(t\right)\propto {\left|\sum_{n=-30}^{30}E_{n}\left(t\right)\exp\left(i\phi_{n}\right)\right|}^{2},$$

where *φ_n_* is an arbitrary phase for the *n*th-mode. Then, photo-carriers are generated to induce a transient current, *J*(*t*), by applying a bias voltage to the gap. As a result, electromagnetic waves are emitted into free space with an amplitude proportional to the time differential of the transient current, *dJ*(*t*)/*dt*. The electromagnetic waves radiated from the system are regarded here as THz-waves by assuming an input light with a bandwidth of a few THz. When the antenna has a wideband characteristic because of the ultrafast relaxation time of the photo-carriers, the THz-wave, *E*_THz_(*t*), can be represented as

(9) $$E_{THz}\left(t\right)\propto \frac{dJ\left(t\right)}{dt}\propto \frac{dn\left(t\right)}{dt}.$$

The THz-waves are collimated with an off-axis parabolic mirror and can then pass through a sample. Then, using another off-axis parabolic mirror, the transmitted THz-waves are focused onto another photoconductive antenna, of the same kind as the first one, which is also irradiated by the probe light to create photo-carriers, *n*(*t*), in the gap. The photo-carriers flow outside the photoconductive antenna as a transient current in proportion to the gap potential determined by the electric field of the irradiated THz-waves. When the delay time *τ* for the probe light is varied, a cross-correlation trace, *C_m_*(*τ*), between the waveforms of the THz-wave and the photo-carriers is obtained. *C_m_*(*τ*) can be represented by

(10) $$C_{m}\left(\tau \right)=\int \frac{dn\left(t\right)}{dt}n\left(t-\tau -m/f_{\mathrm{mod}}\right)dt,$$

where *m* is an integer. The delay time *τ* ranges from −1/2*f*_mod_ to +1/2*f*_mod_. To obtain the THz-TDS output, the cross-correlation trace is Fourier-transformed as

(11) $${\tilde{C}}_{0}\left(\omega \right)=\int_{-1/2f_{\mathrm{mod}}}^{+1/2f_{\mathrm{mod}}}C_{0}\left(\tau \right)\exp\left(-i\omega \tau \right)d\tau =i\omega {\left|\tilde{n}\left(\omega \right)\right|}^{2}.$$

Here, *m* is set to 0. In the autocorrelation configuration shown in Figure 9, the THz-TDS output is simply expressed as above, which is proportional to the power spectrum of the photo-carriers [16]. 

**Figure 10:**
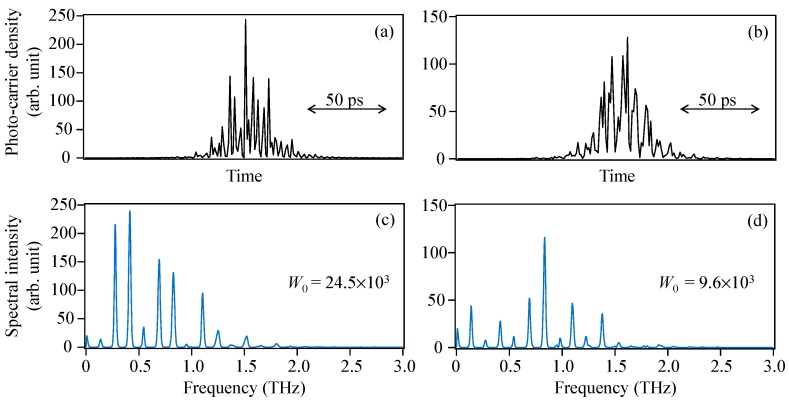
Examples of (**a**); (**b**) single-shot temporal waveforms of the photo-carriers *n*(*t*) and (**c**); (**d**) the corresponding THz-TDS outputs.

**Figure 11:**
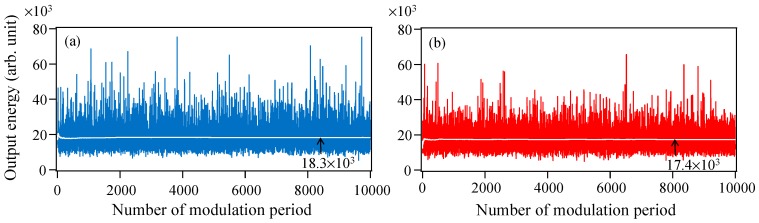
Temporal variations in the THz-TDS outputs from 0 to 10,000 times the modulation period when Langevin noise is (**a**) excluded and (**b**) included. The averaged THz-TDS outputs are indicated by white lines.

First, the THz-TDS output is calculated when Langevin noise is excluded, that is, fully-simultaneous pulse oscillations from a gain-switched multimode semiconductor laser are generated. Figure 10a,b shows examples of single-shot temporal waveforms of the photo-carriers, *n*(*t*), and Figure 10c,d shows the corresponding THz-TDS outputs, $\left|{\tilde{C}}_{0}\left(\omega \right)\right|$. The different phase conditions change the photo-carrier shape drastically even though the total numbers of photo-carriers is constant. The drastic shape change in the photo-carriers is enhanced by the temporal differentiation, and, consequently, the energy of the THz-TDS output, *W*_0_ (=$\underset{\omega }{\sum }\left|{\tilde{C}}_{0}\left(\omega \right)\right|$), varies greatly for every shot, as shown in Figure 10c,d. This is because the Fourier transform of the photo-carriers is not unitary (Parseval’s theorem does not hold for the photo-carriers). Figure 11a shows the temporal variation of *W*_0_ from 0 to 10,000 times the modulation period in the absence of Langevin noise. Although *W*_0_ is varied randomly and considerably from 5400 to 75,000 in arbitrary units, the averaged *W*_0_ nearly converges on a constant value of 18,331 within 2000 times the modulation period. In contrast, in the presence of Langevin noise, a temporal variation of the THz-TDS output, *W*_0_′, is calculated and shown in Figure 11b. The variation in *W*_0_′ ranges from 5000 to 65,000 in arbitrary units and is somewhat suppressed compared with the result in Figure 11a. The averaged *W*_0_′ also becomes nearly constant to 17,305 within 2000 times the modulation period. Although a high-speed scan (1 ns) of the delay line is needed to observe the single-shot of the THz-TDS output, the scan speed in actual experiments is usually much slower than 2000 times the modulation period (2 μs). Hence, the measured value of the THz-TDS output is proportional to the averaged W_0_′. The index of *W*_0_′/*a*^2^*W*_0_ corresponding to *P_rms_*/*P_rms_max_* is estimated to be 95.5% (17,305/(18,331 × 0.994^2^)} × 100), which is the estimated degree of temporal synchronization of the pulse oscillations from a gain-switched multimode semiconductor laser. The decrease in the photo-carrier density in two photoconductive antennae due to the decrease in the input pulse energy in the presence of Langevin noise seen in Figure 5 is taken into account by the coefficient *a*^2^ (*a* = 0.994) in the denominator. 

**Figure 12:**
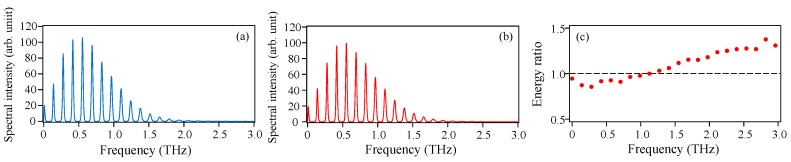
Spectral traces of the THz-TDS outputs averaged over 2000 single-shot spectra, when Langevin noise is (**a**) excluded and (**b**) included; (**c**) ratio of the energy at each line spectrum in (**b**) to that in (**a**).

To check this, the spectral traces of the THz-TDS outputs averaged over 2000 single-shot spectra when Langevin noise is excluded and included are depicted in Figure 12a,b respectively. Corresponding to the results in Figure 11a,b, the spectral shapes of the THz-TDS outputs match each other well. To compare them in detail, the ratio of the energy at each line spectrum in Figure 12b to that in Figure 12a is plotted in Figure 12c. The ratio becomes large toward higher frequencies and is above 1.0 at frequencies higher than 1.2 THz, reflecting the result in Figure 7. Thus, Langevin noise contributes marginally to the improvement of the higher frequency properties of the THz-TDS system. As a result, because the decrease in the THz-TDS output caused by Langevin noise is fairly small (4.5%), an actual gain-switched multimode semiconductor laser is considered to also generate optical pulses simultaneously at all oscillating modes, even when the laser is used in the optical beat detection.

**Figure 13:**
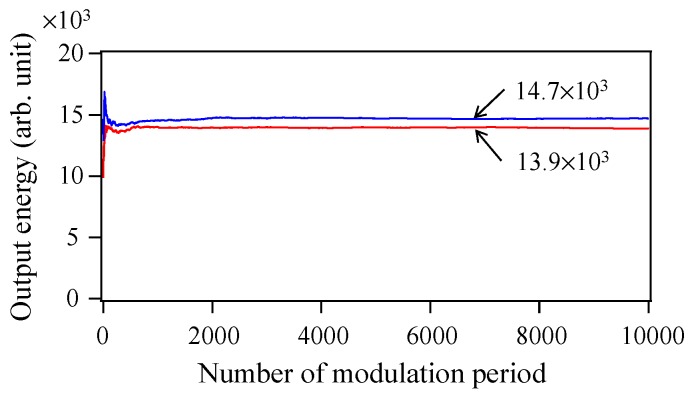
Temporal variations of the averaged THz-TDS outputs from 0 to 10,000 times the modulation period when Langevin noise is excluded (blue line) and included (red line).

Next, we consider the case of *m* = 2 (cross-correlation configuration in Figure 9 in Equation (10). The complex THz-TDS output ${\tilde{C}}_{2}\left(\omega \right)$ is then expressed as

(12) $${\tilde{C}}_{2}\left(\omega \right)=\int_{3/2f_{\mathrm{mod}}}^{5/2f_{\mathrm{mod}}}C_{2}\left(\tau \right)\exp\left(-i\omega \tau \right)d\tau =i\omega \tilde{n}\left(\omega \right){\tilde{n}}^{\prime *}\left(\omega \right),$$

where ${\tilde{n}}^{\prime *}\left(\omega \right)$ is the Fourier transform of *n*(*t −* 2/*f*_mod_). Figure 13 shows temporal variations of the averaged *W*_2_′ (=$\underset{\omega }{\sum }\left|{\tilde{C}}_{2}\left(\omega \right)\right|$) from 0 to 10,000 times the modulation period when Langevin noise is excluded (blue line) and included (red line), respectively. They nearly converge on the constant values of 14,718 and 13,883, respectively, within 2000 times the modulation period. In the case of excluded Langevin noise, the THz-TDS output decreases from 18,331 to 14,718 when *m* is changed from 0 to 2, which indicates a decrease in the coherence degree of the function $\tilde{n}\left(\omega \right)$ under random phase conditions. The coherence degree decreases to 80.3% [(14,718/18,331) × 100], indicating a decrease in the THz-TDS output of 19.7%. In contrast, when the Langevin noise is included, the decrease in the THz-TDS output is 23.3% ({1 − 13,883/(18,331 × 0.994^2^)} × 100), which consists of three contributions, coherence degradation, residual timing jitter, and temporal asynchronization. By eliminating the contribution of the residual timing jitter of 3.9% ((1 − 27.0/28.1) × 100) from the total decrease, the sum of the contributions of the coherence degradation and temporal asynchronization is obtained to be 19.4%, which is almost the same as the contribution of the coherence degradation for *m* = 0. This is reasonable due to the contributions of the coherence degradation and the temporal asynchronization to the decrease in the THz-TDS output balance. For the two cases where the pulse oscillations are fully or partly simultaneous, that is, the oscillating-mode number in a single-shot spectrum is large or small, respectively, the contribution of the coherence degradation is strong and weak, respectively, while the temporal asynchronization contributions are weak and strong, respectively. Thus, the degree of temporal synchronization is masked by the considerable coherence degradation and is found to not be read out from the index of *W_m_*′/*a*^2^*W_m_*, except for the case of *m* = 0. 

**Figure 14:**
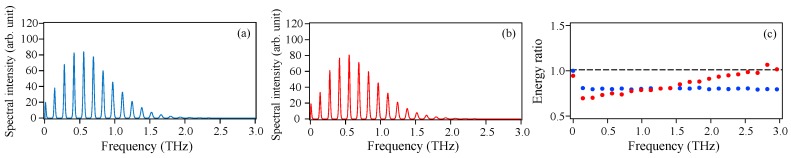
Spectral traces of the THz-TDS outputs averaged over 2000 single-shot spectra, when Langevin noise is (**a**) excluded and (**b**) included and *m* in Equation (10) is set to 2; (**c**) ratio of the energy at each line spectrum in (**a**) (blue circles) and (**b**) (red circles) to that in Figure 12a.

Figure 14 shows the spectral traces of the THz-TDS outputs averaged over 2000 single-shot spectra when Langevin noise is (a) excluded and (b) included and *m* in Equation (10) is set to 2. Compared with the result in Figure 12, the spectral shapes of the traces are found to be almost the same, though the spectral intensities are lower. Figure 14c shows the ratio of the energy at each line spectrum in Figure 14, a (blue circles), b (red circles), to that in Figure 12a. Because the blue circles form a flat line, the contribution of the coherence degradation to the THz-TDS output is found to be frequency-independent. Although it may be possible to estimate the degree of pulse synchronization by taking into account the frequency dependences of the three contributions even for the cases of *m ≠* 0, the method is impractical because of its complexity. The calculation results carried out for different pumping conditions or another wavelength (1550 nm) of the semiconductor laser were confirmed to show a similar tendency to the above obtained results. 

## 5. Conclusions

Using multimode semiconductor laser rate equations that include Langevin noise, the degree of temporal synchronization of the pulse oscillations from a gain-switched multimode semiconductor laser was examined by numerically simulating the output energy of THz time-domain spectroscopy (THz-TDS). The degree was estimated to be 95.5% from the ratio of the averaged THz-TDS output energy in the partly simultaneous oscillating pulse input case (actual) to that in the fully-simultaneous oscillating pulse input case (ideal) under the autocorrelation configuration of the THz-TDS system. This means that the decrease in the THz-TDS output due to the temporal asynchronization of the input pulse oscillations among multi-modes was only 4.5%. Therefore, a gain-switched multimode semiconductor laser can be regarded to equivalently oscillate optical pulses simultaneously at all oscillating modes, in actual applications, even when the laser is used for optical beat detection. It is expected that the degrees of temporal synchronization of chaotic and random optical oscillations from multimode lasers can be examined in a similar way. To conduct related experimental examinations, calibration of the sensitivity of the photoconductive antennae will be necessary.

## Figures and Tables

**Table 1:** Coefficients of parameters in Equation (6).

*j*	*a_j_* (m^3^/s)	*b_j_* (m^−3^)	*c_j_* (m^3^)
0	1.83 × 10^−12^	1.33 × 10^24^	4.92 × 10^−25^
1	4.18 × 10^−14^	9.53 × 10^21^	−1.51 × 10^−27^
2	−8.04 × 10^−16^	2.35 × 10^19^	−4.03 × 10^−29^
3	3.02 × 10^−17^	−1.97 × 10^18^	1.56 × 10^−31^
4	1.60 × 10^−18^	1.76 × 10^17^	6.59 × 10^−33^
5	−2.11 × 10^−19^	1.59 × 10^16^	1.69 × 10^−34^
6	−1.15 × 10^−20^	−1.33 × 10^15^	−2.88 × 10^−35^
7	1.46 × 10^−21^	−6.99 × 10^13^	−7.03 × 10^−37^
8	9.22 × 10^−24^	5.40 × 10^12^	1.11 × 10^−37^
9	−3.71 × 10^−24^	1.12 × 10^11^	−4.95 × 10^−41^
10	3.94 × 10^−26^	−9.49 × 10^9^	−1.56 × 10^−40^
11	3.82 × 10^−27^	−7.78 × 10^7^	9.96 × 10^−43^
12	−6.95 × 10^−29^	7.60 × 10^6^	9.67 × 10^−44^
13	−1.40 × 10^−30^	19,461	−5.57 × 10^−46^
14	3.13 × 10^−32^	−2301	−2.22 × 10^−47^

**Table 2:** Notation and values for parameters [22,23].

Notation	Parameter	Value	Unit
*τ_p_*	Photon lifetime	2.0	ps
*C*_1_	Nonradiative recombination rate	2.0 × 10^8^	s^−1^
*C*_2_	Radiative recombination coefficient	2.0 × 10^−16^	m^3^ s^−1^
*C_3_*	Auger recombination coefficient	0	m^6^ s^−1^
*α*	Linewidth enhancement factor	5.0	
*β*	Spontaneous emission factor	1.0 × 10^−5^	
*ε_S_*	Gain compression factor	0.05 × 10^−23^	m^3^
*δω*/2*π*	Longitudinal mode spacing	0.141	THz
Δ*τ_c_*	Coherence time for amplified spontaneous emission	7.8	ps
*e*	Elementary electric charge	1.60 × 10^−19^	C
*L*	Laser cavity length	300	μm
*V*	Laser cavity volume	180	μm^3^
*n_r_*	Refractive index of active layer	3.6	
*ε*_0_	Dielectric constant for vacuum	8.85 × 10^−12^	F/m
*h* (=2*πћ*)	Planck’s constant	6.63 × 10^−34^	Js
*ω_n_*/2*π*	Central frequency of *n*th mode	375 + 0.141*n*	THz
*I_dc_*	dc-bias current	0.95 × *I*_*th*0_	
*I_mw_*	Amplitude of modulated current	1.90 × *I*_*th*0_	
*I_th0_*	Threshold current for central mode	24	mA
*f_mod_*	Modulation frequency	1.0	GHz
